# Production of Phenolic Compounds and Antioxidant Activity in Hairy Root Cultures of *Salvia plebeia*

**DOI:** 10.3390/plants12223840

**Published:** 2023-11-13

**Authors:** Minsol Choi, Jiwon Yoon, So Hwi Yang, Jae Kwang Kim, Sang Un Park

**Affiliations:** 1 Department of Smart Agriculture Systems, Chungnam National University, 99 Daehak-ro, Yuseong-gu, Daejeon 34134, Republic of Korea; minsolll@cnu.ac.kr; 2 Department of Crop Science, Chungnam National University, 99 Daehak-ro, Daejeon 34134, Republic of Korea; 3 Division of Life Sciences and Convergence Research Center for Insect Vectors, Incheon National University, Incheon 22012, Republic of Korea; thgnltkfkd12@inu.ac.kr

**Keywords:** *Salvia plebeia*, hairy root culture, rosmarinic acid, phenolic compounds, antioxidant activity

## Abstract

*Salvia plebeia* (Lamiaceae) is a medicinal plant containing diverse bioactive constituents that have biological properties. In this study, we determined the optimal conditions (media and auxin) for the hairy root culture of *S. plebeia* for the growth and accumulation of phenolic compounds and evaluated its antioxidant activities. Rosmarinic acid and five phenylpropanoids were detected using high-performance liquid chromatography. The hairy roots grown in 1/2 SH medium with 1 mg/L NAA had a high level of rosmarinic acid content. Hairy roots cultured in 1 mg/L NAA had the highest total content of five phenylpropanoids. Compared to wild-type roots grown in the field, hairy roots (NAA 1) expressed similar levels of rosmarinic acid but significantly enhanced phenylpropanoid accumulation. Furthermore, the total phenolic content and total flavonoid content of hairy roots (NAA 1) were 2.22 and 1.73 times higher than those of wild-type roots. In the results of DPPH, ABTS, and reducing power assays, the hairy roots (NAA 1) showed higher free radical scavenging effects and reduction potential than the wild-type roots. These results suggest that *S. plebeia* hairy roots cultured under optimal conditions, which exhibit enhanced phenolic compound accumulation and antioxidant activity, can potentially be used as sources of antioxidants.

## 1. Introduction

*Salvia plebeia* R. Br., which belongs to the Lamiaceae (Labiatae) family, is a widely distributed medicinal plant in Korea, China, India, Japan, and Australia [1]. This plant, whose common name is ‘Gombo-baechu’ in Korea, has been used as a traditional medicine to treat inflammation and bronchial diseases such as hepatitis, cough, flu, common cold, and hemorrhoids [2]. Phytochemicals in *S. plebeia* have been investigated as natural products that have the pharmacological potential to develop modern drugs and drug intermediates. Previous studies have revealed that *S. plebeia* contains phenolic compounds, triterpenoids, sesquiterpenoids [2], flavonoids [3], and diterpenoids [4]. Additionally, it has various biologically positive activities, including anti-inflammatory, antibacterial, antiviral, anticancer, and antioxidant effects [1,5]. In particular, phenolic compounds from medicinal plants are well known as functional phytochemicals with important pharmaceutical potential, as they have various effects, such as antioxidant, antimicrobial, antiviral, anti-inflammatory, and antipyretic properties [6,7].

Rosmarinic acid, which is a representative phenolic compound of Lamiaceae, was first isolated and identified from rosemary (*Rosmarinus officinalis*). This compound has a strong anti-inflammatory effect and can treat inflammation, such as arthritis, asthma, and atopic dermatitis [8]. In previous studies, rosmarinic acid has been demonstrated to exert powerful antioxidant effects while reducing oxidative stress by increasing the level of antioxidant enzymes [9,10,11,12,13]. Moreover, it is believed that phenolic acids and flavonoids, which are secondary bioactive metabolites found in *S. plebeia*, are considered sources of potent antioxidant activity [5]. Phenolic acids are divided into two major groups: cinnamic acid derivatives (e.g., *p*-coumaric acid, *t*-cinnamic acid, caffeic acid, and ferulic acid) and benzoic acid derivatives (e.g., benzoic acid, *p*-hydroxybenzoic acid, gallic acid, and salicylic acid). Flavonoids are commonly found in diverse medicinal herbs, and quercetin is one of the potent antioxidant phytochemicals [7]. Various medicinal plants contain phenolic compounds, and these phytochemicals are closely related to their antioxidant activities [6,7]. Oxidative stress has been related to many chronic health conditions, such as cancer, heart disease, and diabetes. Antioxidant activity is very effective in inhibiting cell damage and aging, and rosmarinic acid has pharmacological potential in healthcare.

Hairy roots are induced from the sites of plants infected by *Agrobacterium rhizogenes* (*Rhizobium rhizogenes*). Hairy root culture is a suitable biological system for investigating plant metabolic pathways and producing secondary metabolites [14]. The attractive biological properties of this system are known to be rapid root growth, high genetic and biosynthetic stability, and enhanced secondary metabolite production [15]. For these reasons, hairy roots have been considered an appropriate tool for substituting microorganisms, including *Saccharomyces cerevisiae* and *Escherichia coli*, for natural plant metabolite biosynthesis because they are closer to the native host plant [16]. In many studies, the hairy root culture with diverse plants has been used to enhance phytochemical production [17,18,19,20,21].

In several previous studies, *S. plebeia* has been investigated to identify its chemical compositions and has been shown to contain various bioactive compounds with biological activities [1,2,3,4,5]. However, to date, there have been no reports on the hairy root culture of *S. plebeia* induced by *A. rhizogenes* investigating the phenolic compound production and their antioxidant activities. In this study, we aim to induce *S. plebeia* hairy roots to produce pharmacologically important phenolics and to evaluate their antioxidant activities.

## 2. Results

### 2.1. Hairy Root Growth in Different Media and Auxins

**Figure 1:**
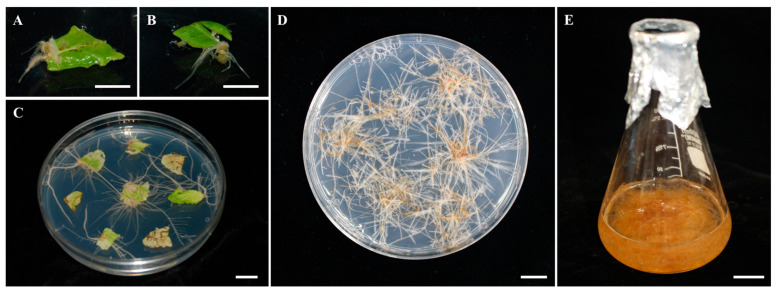
Hairy root induction and cultures of *S. plebeia* using *A. rhizogenes* strain R1000. Hairy roots initiation from a leaf explant after infection (**A**). Development of hairy roots after 7 days (**B**) and 14 days (**C**) from co-cultivation with R1000. Growing hairy roots isolated from an explant and cultured (**D**). Hairy root culture in a flask containing 30 mL of liquid medium (**E**). The scale bar indicates 1 cm.

**Figure 2:**
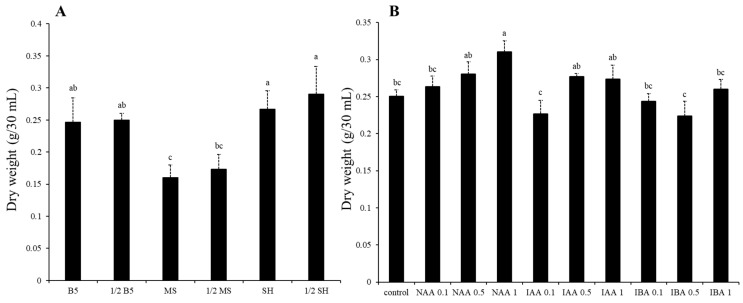
The effect of different media (**A**) and auxin (**B**) on dry weight in *S. plebeia* hairy root. Bars indicate means ± standard deviation with three independent repeats. Different letters represent significant differences according to Tukey’s HSD test (*p* < 0.05).

The growth rate was compared with its dry weight (DW) to investigate the influences of different media and auxin on the growth of *S. plebeia* hairy roots. Hairy roots were cultured in 30 mL of Gamborg (B5), Murashige and Skoog (MS), and Schenk and Hildebrandt (SH) liquid medium at full- and half-strengths (Figure 1). The growth was significantly different depending on the medium type. Hairy roots grown in 1/2 SH medium (0.29 ± 0.04 g DW/30 mL) showed the highest growth rate, followed by those grown in SH medium (0.27 ± 0.03 g DW/30 mL) (Figure 2A). However, hairy roots cultured in 1/2 MS (0.17 ± 0.02 g DW/30 mL) and MS media (0.16 ± 0.02 g DW/30 mL) showed the lowest growth. As a result, 1/2 SH medium was suitable for the growth of *S. plebeia* hairy roots.

Hairy roots were cultured with 30 mL of 1/2 SH liquid medium supplemented with naphthaleneacetic acid (NAA), indoleacetic acid (IAA), and indolebutyric acid (IBA) at three concentrations (0.1, 0.5, and 1 mg/L). To identify the effects of auxin treatments on the growth, the growth rate of hairy roots was measured (Figure 2B). Control hairy roots were cultured in 1/2 SH medium without auxin treatment. The highest growth of hairy roots was 0.31 ± 0.01 g DW/30 mL cultured in the 1/2 SH medium supplemented with 1 mg/L NAA. In all treatments with auxin, the growth of hairy roots increased slightly with increased auxin concentration, except for treatments with IBA. As a result, a 1/2 SH medium with 1 mg/L NAA could be an appropriate condition for the growth of *S. plebeia* hairy roots.

### 2.2. Analysis of Rosmarinic Acid in S. plebeia Hairy Roots

**Figure 3:**
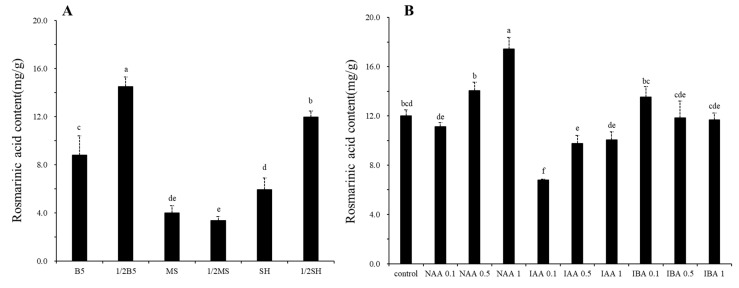
The effect of different media (**A**) and auxin (**B**) on rosmarinic acid content in *S. plebeia* hairy root. Bars indicate means ± standard deviation with three independent repeats. Different letters represent significant differences according to Tukey’s HSD test (*p* < 0.05).

HPLC analysis was performed to investigate the rosmarinic acid accumulation in *S. plebeia* hairy roots cultured in different media types and auxin concentrations. There was a significant difference in rosmarinic acid content depending on the medium type (Figure 3A). The rosmarinic acid content was 14.50, 11.97, 8.80, 5.94, 4.01, and 3.37 mg/g DW in hairy roots cultured in 1/2 B5, 1/2 SH, B5, SH, MS, and 1/2 MS media, respectively. A high level of rosmarinic acid was obtained in hairy roots grown in 1/2 B5 and 1/2 SH, which were 4.3- and 3.55-fold higher than that in the 1/2 MS medium, respectively.

In addition, the rosmarinic acid content was statistically different depending on the treatments of various auxins and their concentrations (Figure 3B). The highest rosmarinic acid content was found in 1 mg/L NAA at 17.44 mg/g DW, which was 1.45-fold higher than that of the control. According to treatments with 0.1, 0.5, and 1 mg/L NAA, rosmarinic acid accumulation in hairy roots increased. However, the rosmarinic acid content was lower in the treatments with IAA than in the control.

### 2.3. Analysis of Phenylpropanoids in S. plebeia Hairy Roots

HPLC analysis was performed to investigate phenylpropanoid accumulation in *S. plebeia* hairy roots cultured in different media types and auxin concentrations. Five phenolic compounds, including caffeic acid, ferulic acid, *p*-hydroxybenzoic acid, *t*-cinnamic acid, and quercetin, were identified and quantified from the *S. plebeia* hairy roots. The highest level of total phenylpropanoids was 49.13 mg/100 g DW in 1/2 SH medium (Table 1). Among these five phenolics in hairy roots, the most abundant compound was quercetin, with a proportion of 73.8–75.2% of the total content, followed by caffeic acid. In the 1/2 SH medium, hairy roots contained the highest levels at 36.28 mg/100 g DW of quercetin and 11.47 mg/100 g DW of caffeic acid.

Furthermore, the phenolic compound accumulation in hairy roots was determined using 1/2 SH medium with several concentrations of three auxins to investigate the effects of auxin on phenylpropanoid biosynthesis. Treatment with 1 mg/L NAA showed the highest total level at 59.55 mg/100 g DW (Table 2). In addition, all auxin treatments showed higher total levels than the control. Quercetin and caffeic acid were observed with ratios of 58.5 to 71.98% and 25.6 to 37.45% of the total content. The highest level of quercetin was 37.56 mg/100 g DW with 1 mg/L IBA. In addition, caffeic acid showed the highest level at 22.3 mg/100 g DW with 1 mg/L NAA. Therefore, considering the growth rate and phenolic content, the optimal conditions for the hairy root culture of *S. plebeia* was 1/2 SH medium supplemented with 1 mg/L NAA.

### 2.4. Comparison of Metabolites in Hairy Roots and Wild-Type Roots of S. plebeia

**Figure 4:**
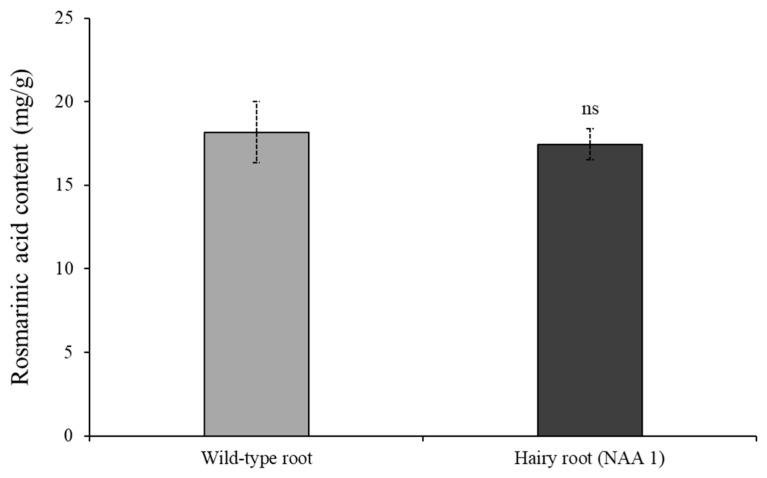
The rosmarinic acid content in the wild-type root of *S. plebeia*. ‘ns’ indicates no significant differences between wild-type root and hairy root (NAA 1) as determined by the *t*-test (*p* < 0.05).

The hairy roots (NAA 1) that were cultured in 1/2 SH medium supplemented with 1 mg/L NAA exhibited the highest level of rosmarinic acid content and were compared with the wild-type roots of *S. plebeia* grown on the field (Figure 4 and Table 3). The rosmarinic acid content in the wild-type roots (18.16 ± 1.82 mg/g DW) was slightly higher than that in the hairy roots (17.44 ± 0.93 mg/g DW). However, there was no significant difference in rosmarinic acid content between hairy roots and wild-type roots. In addition, the accumulation of five phenylpropanoids was compared between the hairy root and the wild-type root. The levels of all phenolic compounds were higher than those in wild-type roots of plants grown in the field, except for *t*-cinnamic acid. In particular, caffeic acid in hairy roots was 2.8-fold higher than that in wild-type roots. The total content of five phenylpropanoids in the hairy roots (59.55 ± 0.08 mg/g DW) was 1.43-fold higher than that in the wild-type roots (41.57 ± 0.53 mg/g DW).

### 2.5. Total Phenolic Content and Total Flavonoid Content

**Figure 5:**
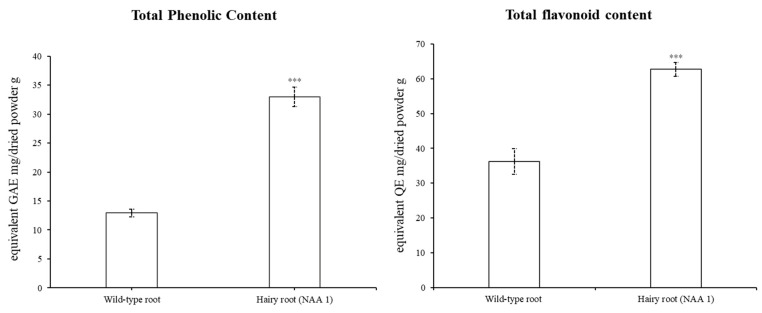
The total phenolic content and total flavonoid content of *S. plebeia* extracts. Asterisks indicate significant differences between the wild-type root and the hairy root (NAA 1) as determined by the *t*-test (*** *p* < 0.001).

The TPC and TFC were measured to compare the wild-type roots and the hairy roots (NAA 1, 1/2 SH medium with 1 mg/L NAA) using a spectrophotometer (Figure 5). The TPC (mg gallic acid equivalent/g dried powder) of hairy roots (32.98 ± 1.68 mg GAE/g) was 2.22-fold higher than that of wild-type roots (12.92 ± 0.67 mg GAE/g). Additionally, the TFC (mg quercetin equivalent/g dried powder) of hairy roots (62.71 ± 1.97 mg QE/g) was 1.73-fold higher than that of wild-type roots (36.28 ± 3.71 mg QE/g). As a result, the TPC and TFC values of the hairy roots (NAA 1) were significantly higher than those of the wild-type roots.

### 2.6. In Vitro Antioxidant Assays

**Figure 6:**
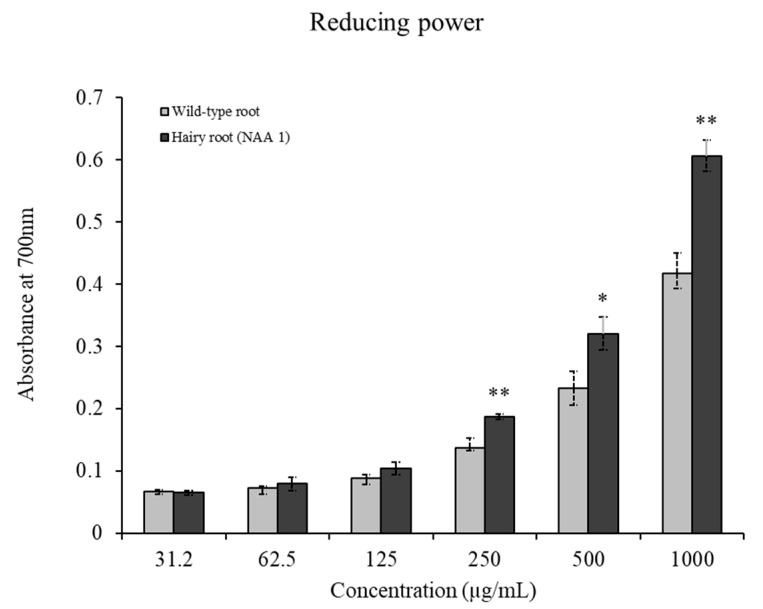
The reducing power of *S. plebeia* extracts. Asterisks indicate significant differences between wild-type root and hairy root (NAA 1) as determined by the *t*-test (* *p* < 0.05; ** *p* < 0.01).

ABTS, DPPH, and reducing power assays were used to evaluate the antioxidant activities of the hairy roots of *S. plebeia* extracts compared with wild-type roots (Table 4 and Figure 6). The hairy roots (NAA 1) showed significantly higher free radical scavenging effects than the wild-type roots in the DPPH and ABTS assays, with IC_50_ values of 96.32 ± 6.45 µg/mL and 354.92 ± 18.7 µg/mL, respectively. In addition, reducing power measured by absorbance at 700 nm showed higher values in the hairy roots (NAA 1) and increased with the sample concentration. Specifically, at 1000 µg/mL, the values of the hairy roots (NAA 1) and the wild-type roots were 0.61 ± 0. 03 and 0.42 ± 0.03, respectively. These results indicate that the hairy roots of *S. plebeia* cultured in 1/2 SH medium supplemented with 1 mg/L NAA have potential as a source of antioxidants with enhanced free radical scavenging activity.

## 3. Discussion

In this study, *S. plebeia* hairy roots were transformed by infection of *A. rhizogenes* strain R1000 using leaf parts from plants grown in aseptic conditions. Hairy root cultures of *S. plebeia* produced rosmarinic acid; concentration range from 3.36 mg/g to 14.50 mg/g according to the media types. Similarly, *Coleus blumei* hairy roots inoculated with *A. rhizogenes* A4 produced rosmarinic acid (50 mg/g) cultured in liquid MS medium without hormone [22]. Chena et al. [23] reported that *Salvia miltiorrhiza* hairy roots, which were transformed using *A. rhizogenes* strain ATCC 15834, produced rosmarinic acid (2.97% DW). Furthermore, hairy root cultures of *Coleus forskohlii* infected by *A. rhizogenes* MAFF 03-01724 produced the highest rosmarinic acid accumulation (4.4 mg/20 mL) when cultured in liquid B5 medium [24]. Additionally, *Dracocephalum moldavica* L. hairy roots generated by *A. rhizogenes* A4 reached the best production in 1/2 B5 medium (78 mg/g dry wt.) under photoperiod conditions [25].

In our results, the hairy root lines cultured in the liquid medium supplemented with each auxin at several concentrations produced rosmarinic acid. In the NAA and IAA treatments, the levels of rosmarinic acid increased with the auxin concentration but slightly decreased upon increasing the IBA concentration. Furthermore, the content in the IAA treatments was lower than in the non-transgenic roots. Similarly, on the one hand, a previous study reported that auxins enhanced rosmarinic acid production in *Nepeta cataria* L. hairy roots [26]. In treatments with IBA and NAA, the rosmarinic acid accumulation of *N. cataria* hairy roots increased with auxin concentrations of 0 to 0.5 mg/L but decreased at 1 mg/L. In addition, the accumulation of rosmarinic acid increased with an increase in the IAA concentration (0–1 mg/L). On the other hand, rosmarinic acid production is reduced in *Lavandula angustifolia* hairy roots when the IBA concentration increases [27]. Specifically, rosmarinic acid content in hairy roots grown with 1 mg/L IBA (0.58 mg/g DW) showed lower content than in the control hairy roots (1.86 mg/g DW). These studies suggest that the effect of auxin on rosmarinic acid accumulation in hairy roots might depend on certain types and concentrations of auxins and plant species.

The hairy root line (NAA 1) had significantly higher values of TPC and TFC than the wild-type roots grown in the field. Previously, several studies reported that hairy root cultures had higher levels of TPC and TFC than non-transformed normal roots, which were not genetically transformed. For instance, Chung et al. [28] reported that *Brassica rapa* ssp. *rapa* (turnip) showed higher TPC and TFC (287.89 mg/g and 4.79 mg/g) in hairy roots than in non-transgenic normal roots. In addition, Korean *Momordica charantia*, Indian *M. charantia* hairy roots [29], and *Ligularia fischeri* Turcz. (Nakai) [30] hairy roots produced higher TPC and TFC values than non-transformed roots. Similarly, the non-transformed roots of *Solanum trilobatum* L. had lower TPC and TFC compared to hairy roots elicited and not elicited with methyl jasmonate [31]. Furthermore, a previous study reported other *Salvia* species; the highest level of TPC in *Salvia bulleyana* hairy roots infected by *A. rhizogenes* strain A4 was 4-fold higher than in normal roots of plants grown under field conditions [32]. 

The results obtained in this study revealed that hairy roots (NAA 1) exhibited stronger antioxidant effects in DPPH, ABTS, and reducing power assays compared to non-transformed roots. Consistently, previous studies that investigated hairy roots of other plant species have reported that hairy roots improved the values of TPC and TFC and possessed stronger antioxidant activities than the roots of plants grown in the field. For example, hairy root cultures of *Dracocephalum moldavica* [25], *S. trilobatum* L. [31], *Momordica dioica* (spine gourd) [33], and *Cucumis angura* [34] had more potential for antioxidant effects compared to non-transgenic roots. Furthermore, hairy root cultures of *Lactuca serriola* L. enhanced the production of phenolics and flavonoids and showed a 31.6–50% increase in DPPH results in comparison to non-transformed roots [35]. Therefore, this study suggests that *S. plebeia* hairy roots can be a useful source for producing various bioactive phenolic compounds to enhance antioxidant activity.

## 4. Materials and Methods

### 4.1. Plant Materials

The seeds of *Salvia plebeia* R. Br. (Lot No. 120 02C006E0) were purchased from Danong Co., Ltd. (Namyangju, Gyeonggi-do, Republic of Korea). Seeds of *S. plebeia* were sterilized with 80% (*v*/*v*) ethanol for 1 min and washed twice with deionized water. Subsequently, the seeds were swirled in 2% (*v*/*v*) sodium hypochlorite solution for 8 min and washed 6 times with sterilized distilled water. Rinsed seeds were dried on sterilized paper and incubated in 25 mL of 1/2 SH basal medium (pH 5.8). The seeds germinated in a growth chamber were maintained at 25 °C in a 16 h light/8 h dark photoperiod for 3 weeks. The seedlings were transferred to plant culture bottles containing the same medium and grown for three months.

### 4.2. Hairy Root Induction and Cultures Using A. rhizogenes

Hairy roots were induced from leaf parts of *S. plebeia* infected with *A. rhizogenes* strain R1000 (OD_600_ = 0.6). The explants were incubated with a bacterial suspension and co-cultured on a 1/2 SH medium for 2 days. They were rinsed with antibiotics solution (300 mg/L cefotaxime) for 1 min and washed 6 times using sterilized deionized water. Subsequently, these explants were dried on sterilized paper and incubated on 1/2 SH medium supplemented with 500 mg/L cefotaxime for 2 weeks. Isolated *S. plebeia* hairy roots were subcultured on cefotaxime (250 mg/L)-supplemented 1/2 SH medium. For experimentation, the grown hairy roots were transferred to three types of liquid media (B5, MS, and SH) with two concentrations (full-strength and half-strength). Additionally, a liquid 1/2 SH medium with several concentrations (0.1, 0.5, and 1 mg/L) of NAA, IAA, and IBA was used for hairy root cultures. The 4 g fresh weight of hairy roots were cultured on a shaker at 100 rpm and 25 °C in the dark for 3 weeks. For the preparation of the samples, the hairy roots were determined in the g DW/30 mL and ground to fine powder. All experiments were performed with three replications.

### 4.3. Preparation of S. plebeia Extracts

The dried powder of the hairy roots and wild-type roots was used for extraction. Of the sample, 100 mg was extracted with 3 mL of aqueous methyl alcohol (80%, *v*/*v*) and vortexed briefly. These samples were sonicated in a water bath for 60 min and centrifuged at 12,000 rpm for 15 min. The extracts were filtered through a syringe filter (0.45 µm) and used for HPLC analysis, determination of the TPC and TFC, and in vitro antioxidant assays.

### 4.4. HPLC Analysis of Rosmarinic Acids

Rosmarinic acid was analyzed using an HPLC system with a Prontosil 120-5-C18-SH column and separated with 0.5% acetic acid in water (eluent A) and acetonitrile (eluent B). The column temperature was controlled at 25 °C and 10 μL of extracts were injected. The gradient program was as follows: 0–10 min: 85–60% A; 10–21 min: 60–35% A; 21–26 min: 35–20% A; 26–28 min: 20–85% A; and 28–38 min: 85% A. The detection was carried out at a wavelength of 342 nm, and the flow rate was 1.0 mL/min. Rosmarinic acid (≥97% purity) was purchased as a standard (Sigma-Aldrich Co., Ltd., St. Louis, MO, USA).

### 4.5. HPLC Analysis of Phenylpropanoids

The phenolic compounds were determined using an NS-4000 HPLC system (Futecs Co., Daejeon, Republic of Korea) with an OptimaPak C_18_ column (RStech; Daejeon, Republic of Korea). The gradient program and conditions were previously described by Park et al. [36]. Phenylpropanoids were determined by comparing the retention time and the spiking test. Concentrations were calculated using respective calibration curves. Quantification of all phenylpropanoids (Sigma-Aldrich Co., Ltd.) was as follows: caffeic acid (≥98%), ferulic acid (≥99%), *t*-cinnamic acid (≥97%), *p*-hydroxybenzoic acid (≥99%), and quercetin (≥95%).

### 4.6. Determination of Total Phenolic and Flavonoid Contents

The dilution concentration of all sample extracts was adjusted to 5000 ppm. The contents were measured according to the protocol described by Park et al. [37] with a slight modification. The absorbance was detected using a spectrophotometer (SPECTROstar Nano, BMG LABTECH, Ortenberg, Germany). The equivalent calibration curve of gallic acid (y = 0.0012x + 0.0558, R^2^ = 0.998) was used for TPC quantification. The TFC in the samples was quantified using the equivalent calibration curve of quercetin (y = 0.002x + 0.0839, R^2^ = 0.9996).

### 4.7. Determination of DPPH Free Radical Scavenging

To analyze the DPPH radical scavenging, sample extracts were prepared with a concentration ranging from 0 to 1000 ppm. Then, 100 µL of 0.2 mM DPPH solution and 100 µL of each concentration extract were mixed. In the sample blank, 100% methyl alcohol that was dissolved in the DPPH solution was added instead of 0.2 mM DPPH. The mixtures were incubated in the dark. After 30 min, the absorbance was detected at 517 nm using a SPECTROstar Nano. The data were calculated as a scavenging effect using the equation reported in a previous study [38]. We used a plotted curve to calculate the 50% inhibitory concentration of 0.2 mM DPPH (IC₅₀) and expressed the results in µL/mL.

### 4.8. Determination of ABTS Free Radical Scavenging

To analyze ABTS radical scavenging, the ABTS buffer solution was prepared immediately before the experiment. The 7 mM ABTS buffer was dissolved in 2.5 mM potassium persulfate buffer and incubated for 16 h in the dark. Finally, the buffer was diluted with distilled water, and the absorbance was checked at 764 nm for an optical density (OD) of 0.7. After the ABTS solution was prepared, 40 µL of each sample extract with a concentration ranging from 0 to 1000 ppm was mixed with 160 µL of the ABTS solution. The absorbance was measured immediately at 734 nm, and the results were calculated in the same way as the DPPH assay. All absorbances were measured using a SPECTROstar Nano microplate reader.

### 4.9. Determination of Reducing Power

Each diluted extract (300 µL) ranging from 31.25 to 10,000 ppm was mixed with 300 µL of sodium phosphate buffer (0.2 M, pH 6.6) and 1% potassium ferricyanide (C₆N₆FeK₃, *w*/*v*). The mixtures were then incubated for 20 min at 50 °C. Following incubation, 300 µL of 10% TCA (trichloroacetic acid, *w*/*v*) buffer was added to the mixture. These mixtures were centrifuged for 10 min at 10,000× *g* rpm. Subsequently, a volume of 500 µL of the supernatant was transferred into a glass test tube containing 500 µL of deionized water and 100 µL of 0.1% ferric chloride (FeCl₃, *w*/*v*). The absorbance was determined at 700 nm using a SPECTROstar Nano microplate reader.

### 4.10. Statistical Analysis

All values were indicated as the mean ± standard deviation in triplicate. Statistical analyses were performed with SPSS Statistics 26.0 (IBM Corporation, New York, NY, USA) using analysis of variance (ANOVA) and a *t*-test. Post hoc tests were applied using Tukey’s HSD or Dunnett’s T3 multiple range tests, depending on whether homoscedasticity was assumed by Levene’s test (*p* < 0.05). Data, which confirmed the homogeneity of variances, assumed equal variances and were analyzed using one-way ANOVA and Tukey’s HSD test. However, data that were not assumed to have equal variances were analyzed by the Brown–Forsythe and Welch ANOVA with Dunnett’s T3 test.

## 5. Conclusions

This study revealed the potential of *S. plebeia* hairy roots, which could be used as a good biomaterial source of antioxidants based on their phenolic compound content and biological activities. The optimal conditions for rapid growth and phenolic compound production in the hairy roots of *S. plebeia* were investigated. As a result, the 1/2 SH liquid medium supplemented with 1 mg/L NAA is considered suitable for growth and phenolic compound accumulation in *S. plebia* hairy roots. Interestingly, hairy roots had significantly higher TPC and TFC levels than field-grown *S. plebeia* roots. Furthermore, the antioxidant activities of hairy roots cultured under appropriate conditions obviously increased compared to those of roots grown in the field. Overall, *S. plebeia* hairy roots cultured for phenolic compound production and rapid growth showed superior antioxidant activity compared to field-grown *S. plebeia* roots.

## Figures and Tables

**Table 1:** The phenylpropanoid content (mg/100 g DW) in the hairy roots of *S. plebeian* cultured in different types of medium.

	*p*-Hydroxybenzoic Acid	Caffeic Acid	Ferulic Acid	*t*-Cinnamic Acid	Quercetin	Total
B5	2.39 ± 0.27 ^a,1^	7.44 ± 0.16 ^de^	1.34 ± 0.28 ^ab^	0.41 ± 0.03 ^a^	35.35 ± 0.13 ^b^	46.93 ± 0.6 ^b^
1/2 B5	1.98 ± 0.08 ^b^	8.48 ± 0.25 ^c^	0.47 ± 0.29 ^ab^	0.32 ± 0.05 ^abc^	35.11 ± 0.13 ^b^	46.35 ± 0.25 ^bc^
MS	2.72 ± 0.04 ^a^	7.26 ± 0.17 ^e^	1.12 ± 0.08 ^a^	0.25 ± 0.01 ^b^	35.38 ± 0.29 ^b^	46.73 ± 0.16 ^b^
1/2 MS	1.83 ± 0.09 ^b^	7.81 ± 0.05 ^d^	1.46 ± 0.17 ^a^	0.16 ± 0 ^c^	34.12 ± 0.18 ^c^	45.38 ± 0.2 ^c^
SH	0.89 ± 0.14 ^c^	8.99 ± 0.19 ^b^	0.11 ± 0.04 ^b^	0.15 ± 0.04 ^bc^	35.24 ± 0.07 ^b^	45.39 ± 0.39 ^c^
1/2 SH	1.1 ± 0.09 ^c^	11.47 ± 0.11 ^a^	0.09 ± 0.02 ^b^	0.19 ± 0.02 ^bc^	36.28 ± 0.28 ^a^	49.13 ± 0.41 ^a^

^1^ Different letters a–e indicate significant differences at *p* < 0.05 according to Tukey’s HSD or Dunnett’s T3 multiple range test. Post hoc tests were applied depending on whether the homoscedasticity was assumed.

**Table 2:** The phenylpropanoid content (mg/100 g DW) in the hairy roots of *S. plebeian* cultured in 1/2 SH medium with different auxin concentrations.

	*p*-Hydroxybenzoic Acid	Caffeic Acid	Ferulic Acid	*t*-Cinnamic Acid	Quercetin	Total
Control	1.16 ± 0.14 ^bc,1^	14.42 ± 0.13 ^d^	0.2 ± 0.06 ^bc^	0.05 ± 0.02 ^c^	36.08 ± 0.1 ^cd^	51.9 ± 0.14 ^f^
NAA 0.1	0.97 ± 0.13 b^cd^	17.47 ± 0.52 ^bc^	0.57 ± 0.34 ^abc^	0.13 ± 0.07 ^abc^	35.8 ± 0.15 ^d^	54.93 ± 0.59 ^bcdef^
NAA 0.5	0.86 ± 0.13 ^def^	17.86 ± 0.2 ^b^	0.96 ± 0.07 ^a^	0.13 ± 0.04 ^abc^	35.13 ± 0.09 ^e^	54.94 ± 0.18 ^b^
NAA 1	1.24 ± 0.06 ^b^	22.3 ± 0.05 ^a^	1.02 ± 0.07 ^a^	0.14 ± 0.03 ^bc^	34.84 ± 0.14 ^e^	59.55 ± 0.08 ^a^
IAA 0.1	1.73 ± 0.15 ^a^	15.68 ± 0.3 ^cd^	0.92 ± 0.06 ^a^	0.28 ± 0.02 ^a^	36.67 ± 0.23 ^b^	55.28 ± 0.55 ^bc^
IAA 0.5	1.01 ± 0.04 ^bcd^	14.44 ± 0.12 ^d^	0.29 ± 0.07 ^bc^	0.26 ± 0.07 ^abc^	36.41 ± 0.05 ^bc^	52.43 ± 0.13 ^ef^
IAA 1	0.67 ± 0.07 ^ef^	16.27 ± 0.14 ^c^	0.38 ± 0.04 ^b^	0.11 ± 0.01 ^bc^	35.95 ± 0.11 ^d^	53.38 ± 0.15 ^cd^
IBA 0.1	0.58 ± 0.06 ^f^	15.95 ± 0.05 ^c^	0.5 ± 0.07 ^bc^	0.13 ± 0.01 ^bc^	36.69 ± 0.18 ^b^	53.84 ± 0.11 ^c^
IBA 0.5	0.89 ± 0.09 ^cde^	14.38 ± 0.14 ^d^	0.37 ± 0.1 b^c^	0.21 ± 0.03 ^ab^	37.28 ± 0.03 ^a^	53.14 ± 0.18 ^cde^
IBA 1	0.81 ± 0.07 ^def^	13.36 ± 0.09 ^e^	0.16 ± 0.03 ^c^	0.29 ± 0.02 ^a^	37.56 ± 0.13 ^a^	52.18 ± 0.25 ^def^

^1^ Different letters a–f indicate significant differences at *p* < 0.05 according to Tukey’s HSD or Dunnett’s T3 multiple range test. Post hoc tests were applied depending on whether the homoscedasticity was assumed.

**Table 3:** The phenylpropanoid content (mg/100 g DW) in the wild-type roots and hairy root of *S. plebeia* treated with 1 mg/L NAA.

	Wild-Type Root	Hairy Root (NAA 1)
*p*-hydroxybenzoic acid	0.83 ± 0.07	1.24 ± 0.06 **
Caffeic acid	7.9 ± 0.35	22.3 ± 0.05 ***
Ferulic acid	0.26 ± 0.03	1.02 ± 0.07 ***
*t*-cinnamic acid	0.33 ± 0.05 **	0.14 ± 0.03
Quercetin	32.24 ± 0.4	34.84 ± 0.14 ***
Total	41.57 ± 0.53	59.55 ± 0.08 ***

Asterisks indicate significant differences between wild-type root and hairy root as determined by the *t*-test (** *p* < 0.01; *** *p* < 0.001).

**Table 4:** Antioxidant activities of *S. plebeia* with DPPH and ABTS assays.

	DPPH (IC50, µg/mL)	ABTS (IC50, µg/mL)
Wild-type root	185.87 ± 34.05	605.86 ± 62.02
Hairy root (NAA 1)	96.32 ± 6.45 *	354.92 ± 18.7 **

Asterisks indicate significant differences between wild-type root and hairy root (NAA 1) as determined by the *t*-test (* *p* < 0.05; ** *p* < 0.01).

## Data Availability

Data are contained within the article.
